# Exploring the question of financial incentives for training amongst non-adopters of MOUD in rural primary care

**DOI:** 10.1186/s13722-022-00353-y

**Published:** 2022-12-14

**Authors:** Treah Haggerty, Nicholas A. Turiano, Tyra Turner, Patricia Dekeseredy, Cara L. Sedney

**Affiliations:** 1 Department of Family Medicine, West Virginia University, 2nd Floor HSS, Morgantown, WV 26506 USA; 2 Department of Psychology, West Virginia Prevention Research Center, West Virginia University, Morgantown, WV 26506 USA; 3 Health Sciences, West Virginia University, Morgantown, WV 26506 USA; 4 Department of Neurosurgery, West Virginia University, Morgantown, WV 26506 USA

**Keywords:** Opioid, Rural, MOUD, Training, Opioid use disorder, Opioid treatment provider

## Abstract

**Background:**

Medication for opioid use disorder (MOUD) includes administering medications such as buprenorphine or methadone, often with mental health services. MOUD has been shown to significantly improve outcomes and success of recovery from opioid use disorder. In WV, only 18% of providers including physicians, physician assistants, and nurse practitioners are waivered, and 44% of non-waivered providers were not interested in free training even if compensated. This exploratory research seeks to understand intervention-related stigma in community-based primary care providers in rural West Virginia, determine whether financial incentives for training may be linked to levels of stigma, and what level of financial incentives would be required for non-adopters of MOUD services provision to obtain training.

**Method:**

Survey questions were included in the West Virginia Practice-Based Research Network (WVPBRN) annual Collective Outreach & Research Engagement (CORE) Survey and delivered electronically to each practice site in WV. General demographic, staff attitudes and views on compensation for immersion training for delivering MOUD therapy in primary care offices were returned. Statistical analysis included logistic and multinomial logistic regression and an independent samples t-test.

**Results:**

Data were collected from 102 participants. Perceived stigma did significantly predict having a waiver with every 1-unit increase in stigma being associated with a 65% decreased odds of possessing a waiver for buprenorphine/MOUD (*OR* = 0.35; 95% CI  0.16–0.78, *p* = 0.01). Further, t-test analyses suggested there was a statistically significant mean difference in perceived stigma (*t*(100) = 2.78, *p* = 0.006) with those possessing a waiver (*M* = 1.56; *SD* = 0.51) having a significantly lower perceived stigma than those without a waiver (*M* = 1.92; *SD* = 0.57). There was no statistically significant association of stigma on whether someone with a waiver actually prescribed MOUD or not (*OR* = 0.28; 95% CI  0.04–2.27, *p* = 0.234).

**Conclusion:**

This survey of rural primary care providers demonstrates that stigmatizing beliefs related to MOUD impact the desired financial incentive to complete a one-day immersion, and that currently unwaivered providers endorse more stigmatizing beliefs about MOUD when compared to currently waivered providers. Furthermore, providers who endorse stigmatizing beliefs with respect to MOUD require higher levels of compensation to consider such training.

## Introduction

Opioid use disorder (OUD) is a prime public health concern this decade, with over 1.9 million people reporting OUD [1]. Medication for opioid use disorder (MOUD) includes administering medications such as buprenorphine or methadone [2], often with mental health services [3]. MOUD has been shown to significantly improve outcomes and success of recovery from OUD [4], which is seen as a lifelong, chronic illness [5]. There is a negative correlation between the use of opioid agonists and overdose deaths, with one study showing a 59% decrease in drug overdose deaths for those receiving methadone [6]. The Substance Abuse and Mental Health Services Administration (SAMHSA) reports the effectiveness of MOUD has helped patients maintain positive life changes and lowers the risk of relapse [7]. Patient improvements in psychological well-being and social integration from MOUD have also been reported [8]. Although the benefits of MOUD are evidenced-based, the adoption of MOUD is still lagging, leaving this treatment unavailable to large numbers of patients who would benefit from it [9]. This effect is particularly severe in rural areas, including West Virginia, where only 18% of providers possess a waiver to prescribe buprenorphine [10].

In parts of Ohio, regional challenges such as conservative culture and discordance among health professionals are barriers that contribute to the stigma against MOUD [11]. In rural New Mexico, the ECHO (Extension for Community Healthcare Outcomes) model has previously been used to address barriers, such as stigma, to MOUD use in primary care clinics [12]. The framework utilized telecommunication methods to educate providers on the use of MOUD but showed a disconnect between the need for treatment and the integration of services. Participants reported barriers to the intervention, including time constraints due to demanding work schedules and lack of instrumental support from clinic staff. Importantly, attitudinal problems and workforce misunderstandings/stigma regarding the nature of MOUD have been identified as a significant negative determinant of the adoption of MOUD in rural areas [13].

There are similar barriers in West Virginia (WV). West Virginia is a rural state in the Appalachian mountain range. In 2015 the state is estimated to have 1319 primary care physicians [14, 15]. At this time the state population is around 1.8 million. There are 31 community health centers with about 180 sites. In a state survey of Medicaid-enrolled providers, although only 18% of providers, including physicians, physician assistants, and nurse practitioners, are waivered, 84% of waivered providers actually provided MOUD. This is well above the national average [10]. However, 44% of non-waivered providers were not interested in free training even if compensated [10]. These respondents identified reasons such as "substituting one addiction for another", “refuse to be an enabler". “abstinence is the best policy“, and “not proven safe or effective” as reasons for their lack of interest [10]. While contact interventions have shown promise in altering stigma associated with MOUD and influencing the pre-contemplation stage of change, the long-term effectiveness of such interventions is unknown.

The lack of adoption for MOUD may be linked to stigma, as it has been suggested that this MOUD delivery carries a separate stigma amongst both patients and physicians as compared to substance use itself [16]. Although the treatment of OUD can be complex, it is necessary to understand provider level intervention-related, or MOUD delivery, stigma to increase MOUD uptake ultimately. This exploratory research seeks to understand intervention-related stigma to provider MOUD delivery in community-based primary care providers in rural West Virginia, determine whether financial incentives for training in primary care MOUD delivery may be linked to levels of stigma, and what level of financial incentives would be required for non-adopters of MOUD services provision to obtain training.

## Methods

Objective: The objective of this work was to understand how stigma is associated with desired levels of compensation for a one-day immersive experience in a primary care-based MOUD clinic amongst primary care providers who do or do not want to become waivered. We additionally sought to understand how unsupportive staff attitudes were reflected in perceived barriers to prescribing MOUD. The West Virginia University Institutional Review Board approved this study.

### Survey participants

Data were collected using two modalities—online through the REDCap survey system (N = 68) and Zoom polling (N = 34). For purposes of this paper, responses were merged for a final sample of 102 participants. 1 The Collective Outreach & Research Engagement (CORE) Survey is delivered to contacts identified in each practice site or collaborator in the West Virginia Practice-Based Research Network (WVPBRN). A snowball convenience sampling plan was used. WVPBRN contacts were encouraged to share the survey within their health system. The survey link was emailed to the primary contacts three times between March 25th, 2020 and May 1st, 2020. These results were augmented in a WVPBRN member meeting where electronic zoom survey data was collected on the survey results.

The WVPBRN is a group of WV primary care clinicians and practices that partner in research to identify and answer pragmatic research questions. The WVPBRN is comprised of 72 Federally Qualified Health Centers, two Rural Health Centers, 20 academic-based centers, and one critical access hospital [17].

### Survey instrument development

Survey questions were developed by authors C.S. and T.H. to be included in the West Virginia Practice-Based Research Network’s (WVPBRN) omnibus survey titled the Collective Outreach Engagement (CORE) survey [18]. This omnibus survey includes up to 10 questions per submitted and accepted proposal. This survey is delivered electronically utilizing REDCap annually.

The survey instrument contained general demographic information relevant to MOUD and the “unsupportive staff attitudes” component of a previously validated instrument [19] to assess the level of stigma. The survey included questions that related to participants’ views on compensation for immersion training in delivering MOUD therapy in primary care offices. Participants were able to skip questions in the survey.

### Study variables

The key predictor for the current study was the mean score on 5-items assessing the perceived level of stigma regarding addiction and treatment options (Table 1). Participants were asked to respond to each question on a 1 (strongly disagree) to 4 (strongly agree) scale. An average score was then created, with higher scores representing greater perceived stigma.

**Table 1:** Descriptive statistics for stigma items

	M (SD)	Range
Medications for treating substance abuse are inconsistent with this center’s treating philosophy (N = 102)	1.74 (0.69)	1–4
There is not enough evidence that substance abuse treatment medications are clinically effective (N = 101)	1.78 (0.58)	1–3
There are better alternatives to using medications as part of substance abuse treatment (N = 102)	2.19 (0.69)	1–4
Using medications to treat addiction is substituting one drug addiction for another (N = 67)	2.07 (0.74)	1–4
Our patients are not interested in using medications as part of their substance abuse treatment plans (N = 66)	1.62 (0.49)	1–2
Average	1.87 (0.52)	0.40–3.00

Several outcomes were utilized in the current study. To determine whether a participant had a waiver to prescribe buprenorphine/MOUD, they answered *yes* or *no* to the following question: “*Do you currently possess a waiver for buprenorphine/MAT*?” For those who reported that they did possess a waiver, they were asked the following question with a *yes/no* response format: “*Do you prescribe buprenorphine/MAT*?”. For those that reported not having a waiver, they were asked whether they would want immersion training using a *yes/no* format: “*Would you be interested in a 1-day immersion in a primary care-based MAT clinic if you and your clinic staff were compensated for time*?”. Lastly, those who did not possess a waiver were asked how much compensation they would need to complete such training: “*What level of monetary incentive would encourage you to consider training, above compensation for time*?”. Responses were as follows: (1) $0–1000; (2) $1001–2000; (3) $2001–5000; (4) $5001–10,000; (5) > $10,000. Participants were also able to select options of *non-monetary incentive* (N = 9) and *not interested* (N = 3). Of note, “MAT” was utilized at the time of survey roll-out but verbiage has been changed to MOUD to be in accordance with current best practice throughout the rest of this paper.

### Research questions and analyses

Since there were a variety of research questions in the current survey, we utilized logistic and multinomial logistic regression and an independent samples t-test. Alpha was set to 0.05 for all analyses. An independent samples t-test was used to determine if there was a mean difference in perceived stigma between those that possess the waiver versus those that do not possess the waiver. A logistic regression was used to evaluate if perceived stigma was associated with currently possessing a waiver for buprenorphine/MOUD and if perceived stigma was associated with actually prescribing buprenorphine/MOUD even if the participant possessed the waiver to do so. Also, is perceived stigma associated with perceived participation in an immersion training. A multinomial logistic regression was used to evaluate if the level of stigma in participants who possess a waiver was associated with the odds of wanting a specific level of compensation to complete immersion training.

## Results

Basic demographic information can be found in Table 2. A statistically significant effect emerged for our first analysis suggesting perceived stigma did significantly predict having a waiver with every 1-unit increase in stigma being associated with an 65% decreased odds of possessing a waiver for buprenorphine/MOUD (*OR* = 0.35; 95% CI  0.16–0.78, *p* = 0.01). Further, t-test analyses suggested there was a statistically significant mean difference in perceived stigma (*t*(100) = 2.78, *p* = 0.006) with those possessing a waiver (*M* = 1.56; *SD* = 0.51) having a significantly lower perceived stigma than those without a waiver (*M* = 1.92; *SD* = 0.57). However, there was no statistically significant effect of stigma on whether someone with a waiver actually prescribed MOUD or not (*OR* = 0.28; 95% CI  0.04–2.27, *p* = 0.234).

Analyses exploring those who did not possess a waiver for buprenorphine/MOUD (N = 79), did not reveal a statistically significant effect (OR = 0.37; 95% CI  0.14–1.04, p = .058). Moreover, when examining preferred compensation levels, every 1-unit increase in stigma was associated with a 207% increased odds of preferring compensation of $5,0001–10,000 (*OR* = 3.065; 95% CI  1.00–9.40; *p* = 0.050), and a 165% increased odds of preferring $10,000 (*OR* = 2.65; 95% CI  1.07–6.56; *p* = 0.035) as compensation for getting the training (as compared to the $0–1,000 referent group. No statistically significant effects were found for the $1001–2000 and $2001–5000 compensation groups.

**Table 2:** Descriptive information

	Mean (SD) or %
Degree	MD/DO/NP/PA = 92% RN/LPN/LCP/PhD = 8%
Employment duration	< 1 year = 10% 1–3 years = 21% 4–6 years = 16% 7–10 years = 16% 11–20 years = 30% 21–30 years = 4% 30 + years = 4%
Possess waiver	No = 76% Yes = 24%
Currently prescribe (only those with waiver)	No = 76% Yes = 24%
Barriers to possessing a waiver (only those without a waiver)	Regulations = 24% Liability/risk to license = 26% Lack of skill set/knowledge = 36% Not financially feasible = 5% Lack of support from employer = 13% Lack of patient need/interest = 15% Other reason = 30%
Interest in immersion training (only those without a waiver)	No = 69% Yes = 31%
Level of compensation (only those without a waiver)	$0–1,000 = 21% $1,001–2,000 = 15% $2,001–5,000 = 16% $5,001–10,000 = 9% > $10,000 = 17% Non-monetary incentive = 12% Not interested = 4%
Perceived stigma	Possess Waiver = 1.56 (0.51) Waivered providers who do not prescribe MOUD = 1.63 (0.55) Waivered providers who do prescribe MOUD = 1.34 (0.26) No Waiver = 1.92 (0.57)

## Discussion

Implementing evidence-based practices (EBPs) into real-world settings is notoriously slow, taking on average 17 years [20]. Often the barriers to implementation are multifactorial. The implementation of MOUD in community settings has been extensively studied, and the reasons for lack of uptake appear to be complex. This mismatch has led to direct study regarding how to increase the uptake of such practices in communities. Because barriers to implementation of MOUD tend to be multifactorial and often include a stigma component, it is possible that financial incentives to complete needed interventions to facilitate MOUD care may be effective. In a study of emergency department providers, researchers found an increase in completion of X waiver training amongst prescribers and an increase in subsequent buprenorphine prescribing amongst those prescribers, after an initiative to offer $750 (about half the normal shift rate for emergency department providers at that facilitate) plus the cost of the training course to complete the needed X waiver training [21].

However, it’s not understood how stigmatizing beliefs may play into the efficacy of financial incentives for training. We sought to understand how stigmatizing beliefs related to MOUD would impact the desired financial incentive to complete a one-day immersion, and found that currently unwaivered providers do endorse more stigmatizing beliefs about MOUD when compared to currently waivered providers, and that providers who endorse stigmatizing beliefs with respect to MOUD require higher levels of compensation to consider such training, with 17% of unwaivered providers choosing “>$10,000” as their preferred financial incentive to complete a one day immersion in MOUD. Importantly, however, only 4% were not interested for any amount. This may be relevant in areas such as WV where uptake of MOUD is low and interest in in becoming waivered is also low, as in our sample where nearly 70% of unwaivered providers expressed no interest in obtaining their waiver. Providing financial incentives for to precontemplative non-adopters of MOUD may be a way of engaging them in immersion experiences and thus addressing stigmatizing beliefs as well as other barriers such as those endorsed by our sample.

Importantly, addressing barriers to MOUD prescription depends on accurate accounting or measuring of those barriers, but this depends upon accurate self-reporting amongst non-adopters. While regulatory burdens have been previously cited as a reason for lack of uptake of MOUD [22], we noted that increased stigma was associated with answering “regulations” as their reason for not prescribing MOUD in our survey data. This is of importance because addressing regulatory barriers alone may not increase the uptake of MOUD if the perception of regulatory barriers is also related to stigmatizing beliefs regarding MOUD.

MOUD-associated stigma is well characterized and is present within multiple stakeholder groups and in multiple contexts. MOUD-associated stigma is seen in 12-step groups [23], physicians [24], other members of the healthcare team [25], patients [26], and the general public [24]. Stigma interventions are the subject of increasing study, and can generally be categorized into educational interventions, contact interventions with stigmatized groups, mixed education/contact, and challenging negative attitudes [27]. Contact and mixed contact-education interventions seem to be the most effective, including with respect to mental health stigma [28, 29], although some studies do not support that assertion [30]. One promising stigma reduction technique for OUD is positive empathy inventions, which has been studied in the form of an imagined positive contact interaction [31].

However, one particularly problematic aspect of stigma regarding MOUD is that stigma itself may prevent interaction with stigma reduction interventions or other interventions aimed at barriers to MOUD care. Therefore, there may be a role for financial incentives to increase this interaction. Financial incentives are widely used to influence physician behavior with respect to productivity and quality. It has also been utilized to increase uptake of specific interventions, for example smoking assessments [32], and additional health screenings [33]. However, because of the multifactorial nature of intervention uptake, financial incentives may not always translate to improved uptake of the desired behavior or health impact; for instance, physician incentive was not effective in reducing readmission [34] or improving patient access to care when physicians were reimbursed for relocation to rural areas [35].

This study does have several limitations worth mentioning. First this was a convenience sample of participants. The results of this study may not be generalizable to other populations, however we feel our methodology may be replicated in other settings. Also, the participants in this study were members of the West Virginia Practice-Based Research Network and therefore may represent a sample biased toward involvement in research. Participant demographics, such as employment duration or practice type, were excluded from the analysis. Future directions could look at provider demographics in relation to MOUD stigma or attainment and use of an MOUS prescription waiver. Lastly, due to the data collection techniques the analysis had to include a mean stigma score that was calculated differently between sampling groups which we explained in the methods.

Evidence does point to survey respondents reporting that they do not have administrative support in applying MOUD in their clinical setting. In the current study waivered providers identified “resistance from practice providers,“ and “resistance from practice administration” as concerns amongst their own practices, and this is consistent with other research from rural areas [10]. Therefore, it seems that MOUD-associated stigma may play a significant role in West Virginia as in other rural locations. Our work suggests that level of stigma may relate to the amount of compensation required by providers to participate in stigma-reduction contact interventions. However, it is not known if incentivizing primary care physicians for MOUD immersion training is feasible or acceptable. Stigma interventions, such as immersion training could be combined with other stigma reducing interventions such as positive empathy inventions which has been found to have positive effects on reducing stigma to individuals with OUD [31]. This work lays the groundwork for design of future stigma reduction interventions in W.V however it is not known which interventions would be best delivered and how. Future research is needed in this area.

## Conclusion

This survey of rural primary care providers demonstrates that stigmatizing beliefs related to MOUD impact the desired financial incentive to complete a one-day immersion, and found that currently unwaivered providers endorse more stigmatizing beliefs about MOUD when compared to currently waivered providers. Furthermore, providers who endorse stigmatizing beliefs with respect to MOUD require higher levels of compensation to consider such training.

## Data Availability

The datasets used and analyzed during the current study are available from the corresponding author on reasonable request.

## Abbreviations

OUD: Opioid use disorder
MOUD: Medication for opioid use disorder
SAMHSA: Substance abuse and mental health services administration
ECHO: Extension for community healthcare outcomes
W.V.: West Virginia
WVPBRN: West Virginia practice-based research network
CORE: Collective outreach engagement
MAT: Medication assisted treatment
EBP: Evidence based practice

## Footnotes

1 Since ZOOM only permits a total of 10 questions, two questions from the stigma scale were not included in the survey (1. Using medications to treat addiction is substituting one drug addiction for another; 2. Our patients are not interested in using medications as part of their substance abuse treatment plans). Thus, the mean stigma score for the 36 participants completing the Zoom survey includes only 3 stigma questions whereas the 81 participants who completed the REDCap survey had a stigma score that included all 5 questions. Sensitivity analyses were conducted where the mean stigma score for the REDCap survey was also set to only the three questions and all analyses were conducted again to ensure robustness. There were no appreciable differences in findings using the 3-question stigma mean score.
